# Fast detection and data compensation for electrodes disconnection in long-term monitoring of dynamic brain electrical impedance tomography

**DOI:** 10.1186/s12938-016-0294-7

**Published:** 2017-01-07

**Authors:** Ge Zhang, Meng Dai, Lin Yang, Weichen Li, Haoting Li, Canhua Xu, Xuetao Shi, Xiuzhen Dong, Feng Fu

**Affiliations:** Department of Biomedical Engineering, Fourth Military Medical University, Xi’an, China

**Keywords:** Brain electrical impedance tomography, Electrode disconnection, Weighted-correlation coefficient, Wavelet transform, Grey model

## Abstract

**Background:**

Electrode disconnection is a common occurrence during long-term monitoring of brain electrical impedance tomography (EIT) in clinical settings. The data acquisition system suffers remarkable data loss which results in image reconstruction failure. The aim of this study was to: (1) detect disconnected electrodes and (2) account for invalid data.

**Methods:**

Weighted correlation coefficient for each electrode was calculated based on the measurement differences between well-connected and disconnected electrodes. Disconnected electrodes were identified by filtering out abnormal coefficients with discrete wavelet transforms. Further, previously valid measurements were utilized to establish grey model. The invalid frames after electrode disconnection were substituted with the data estimated by grey model. The proposed approach was evaluated on resistor phantom and with eight patients in clinical settings.

**Results:**

The proposed method was able to detect 1 or 2 disconnected electrodes with an accuracy of 100%; to detect 3 and 4 disconnected electrodes with accuracy of 92 and 84% respectively. The time cost of electrode detection was within 0.018 s. Further, the proposed method was capable to compensate at least 60 subsequent frames of data and restore the normal image reconstruction within 0.4 s and with a mean relative error smaller than 0.01%.

**Conclusions:**

In this paper, we proposed a two-step approach to detect multiple disconnected electrodes and to compensate the invalid frames of data after disconnection. Our method is capable of detecting more disconnected electrodes with higher accuracy compared to methods proposed in previous studies. Further, our method provides estimations during the faulty measurement period until the medical staff reconnects the electrodes. This work would improve the clinical practicability of dynamic brain EIT and contribute to its further promotion.

## Background

Dynamic brain electrical impedance tomography (EIT) reconstructs the changes in intracranial conductivities at two different instants by injecting safe currents and measuring boundary voltages through 16 or more surface electrodes [1, 2]. Therefore, well-connected electrodes are a prerequisite for normal data acquisition and image reconstruction. However, dynamic brain EIT monitoring is a long-term process. Electrode disconnection is a common occurrence because of several factors such as patient body movement, conscious or unconscious head rotation, and operations by medical staffs [3, 4]. The disconnection affects the quality of acquired data, which gives rise to reconstruction failure. Therefore, it is essential to investigate the case of disconnected electrodes in clinical experiments for improving the applicability of long-term monitoring.

In contrast to other conventional long-term physiological parameter monitors such as electrocardiogram monitors, the electrodes of brain EIT systems are laid under bandages around the transverse plane of the head. Therefore, it is difficult to visually discover the disconnected electrodes. Disconnected electrodes can be detected by improving hardware of EIT systems. However, the hundreds of measuring channels with many possible electrode combinations make redesigning the data acquisition system troublesome. Moreover, such improvements would not help to compensate for invalid data produced by disconnected electrodes. Therefore, a fast and convenient method is needed to discover disconnected electrodes and to compensate for the invalid data using a specific algorithm based on the characteristics of measurement.

Some similar studies have been performed in lung EIT. Adler proposed a methodology that calculated the image with remaining good data by modifying the noise covariance matrix in maximum a priori (MAP) reconstruction algorithm [3]. However, this method requires priori information of the disconnected electrode. Asfaw and Adler realized automatic detection of detached electrodes based on comparisons between measured voltages and simulated voltages, but it is not applicable in real-time [5]. Hartinger et al. [4] presented a detection method for faulty electrodes’ management based on reciprocal principle. This method’s detectability for multiple disconnected electrodes still needs improvement and the application requires extra reciprocity measurement. Nevertheless, the dynamic lung EIT is different from dynamic brain EIT because the imaging interval between the reference data and current data is shorter [6, 7]. Besides, there are other studies of electrode error detection in multi-sensor devices [8–10]. However, their application conditions are not suitable for our brain EIT case. Recent studies of EIT electrodes have primarily concentrated on the impact of electrode–skin contact impedance on EIT image quality, which is a different problem from disconnection [11–14].

In this study, we develop a real-time detection for multiple disconnected electrodes to alert medical staff and to help to fix the disconnected electrodes as soon as possible. And compensation for invalid data is proposed to restore the image reconstruction, which is necessary for medical staff to gain approximate monitoring results while the data acquisition electrodes are disconnected. The novelty of our proposed approach is as follows. Without modifying the data acquisition protocol or the reconstruction algorithm as proposed in previous studies, we presented a measured-data-based approach to deal with the electrode disconnection by two steps. For disconnected electrode detection, we utilized the measured voltages and correlation coefficients to calculate weighted correlated coefficient for each electrode, and distinguished the EVC values corresponding to disconnected electrodes by wavelet transform. Besides, we employed EVC calculation in a circular fashion to unify the EVC calculation environments and simplify complicated scenarios with different number and location of disconnected electrodes into limited cases. In the data compensation, we utilize grey model prediction established by previous good data to replace the lost frames of date.

## Methods

The methodology developed to manage the disconnection of electrodes is described in “Analyzing the influence of electrode disconnection on measurements”, “Calculation of electrode variation coefficient”, “Detection of disconnected electrodes based on wavelet decomposition” and “Compensation algorithm based on grey model method” sections . In “Data acquisition procedure” section, we describe the details about data acquisition and experimental operations.

### Analyzing the influence of electrode disconnection on measurements

In this section, we illustrate the difference between measurements with disconnections and measurements without disconnections via a theoretical analysis and phantom tests.

There are two primary data collection strategies for EIT data acquisition, namely pair-driven and multiple-driven electrode systems. The applied potential tomography (APT) and adaptive current tomography (ACT) systems are corresponding implementations [15]. The typical APT system was developed by Sheffield University [16]. This system attaches 16 electrodes at equal distances around the body surface. Successively applied current is injected through a pair of electrodes, and the voltages between other adjacent noncurrent-carrying electrodes are measured. A frame of data is collected after the procedure is repeated for each adjacent pair. In dynamic imaging, the APT system uses one frame of data as a reference and another frame of measurement data as the current data. In the reconstruction algorithm, if there is any impedance change that leads a variation in the boundary voltage, the internal impedance change will be displayed in the image. The ACT system applies current to all electrodes and simultaneously measures the voltage on all electrodes [17]. For an ACT system with L electrodes, there are L(L − 1)/2 independent measurements, since at most L − 1 independent currents can be applied and the current-to-voltage operator is self-joint [15, 18].

**Fig. 1:**
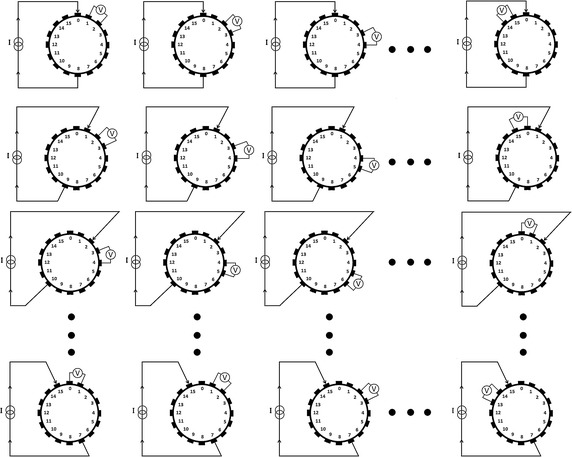
Illustration of opposite-drive adjacent-measurement protocol. Subfigures in the *same row* represent the procedure of measuring with once current excitation. Subfigures in the *same column* represent the excitation switch procedure

Our brain EIT system is an APT system [19]. It applies 16 electrodes on the transverse plane of the patient’s head with opposite-drive adjacent-measurement protocol [20]. The process of boundary voltages acquisition was shown in Fig. 1. When the current was injected through electrode pair 0–8, excluding the measurements from drive electrodes, the 12 sets of voltage differences between electrode pairs 1–2, 2–3, 3–4, 4–5, 5–6, 6–7, 9–10, 10–11, 11–12, 12–13, 13–14, 14–15 were measured. Then the current were sequentially injected through electrode pairs 1–9, 2–10…15–7 and the voltage differences on the other adjacent electrodes were measured. After the completion of 16 excitations, a frame of data was generated with $16 \times (16{-}4) = 192$ valid measurements [21].

**Fig. 2:**
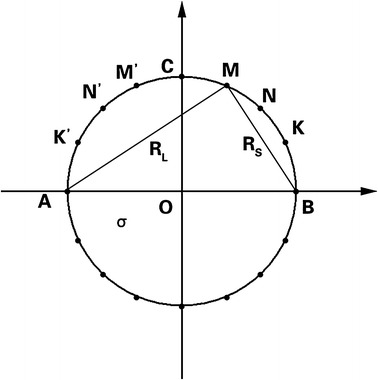
Illustration of the analytical solution of electric potential in a two-dimension homogeneous circular field. Marked points such as *A*, *B*, *M*, *N*, *K* represent electrodes. *σ* is the conductivity and *R* is the distance between electrodes

Here, we assumed an ideal situation of a homogeneous circular medium in a vacuum with 16 electrodes placed on the surface at equal spacing (Fig. 2). The current *I* flows into the circular field through electrode A and flows out through B. Here, we set electrode B as ground to simulate a single-end current source. Then, the analytical solution of the electric potential of the circular point is [22–24]

1 $$ \varphi_{num} = \frac{I}{\pi \sigma }\ln \frac{{R_{L,num} }}{{R_{S,num} }} $$

where *σ* is the conductivity of circular medium, *num* is point index inside the circle, and *R* is the distance from the point and the drive electrode pairs. Then, the potential difference between two points is

2 $$ \varphi_{ij} = \left| {\varphi_{i} - \varphi_{j} } \right| = \frac{I}{\pi \sigma }\left| {\ln \left( {\frac{{R_{L,i} }}{{R_{S,i} }} \cdot \frac{{R_{S,j} }}{{R_{L,j} }}} \right)} \right| $$

Regarding the differential voltage of adjacent electrodes in the first quadrant, the potentials are written as *φ*_*KN*_, *φ*_*NM*_, *φ*_*MC*_, while *φ*_*KN*_ > *φ*_*NM*_ > *φ*_*MC*_. In the second quadrant, $\varphi_{{K^{{\prime }} N^{{\prime }} }} > \varphi_{{N^{{\prime }} M^{{\prime }} }} > \varphi_{{M^{{\prime }} C^{{\prime }} }}$, which is the same as in the opposing two quadrants.

If the positive drive electrode A is disconnected, there will be no current injected into the medium, which is equivalent to *I* = 0. Thus, the potential is 0 everywhere in the circle. If the negative drive electrode B is disconnected, we can only solve the potential distribution via the method of the potential distribution of a point current source in a homogenous field. The potential calculation Eq. 1 can be approximately rewritten as [25]

3 $$ \varphi_{num} = \frac{I}{r\sigma } \cdot A $$

where *r* is the distance from point *num* to the point of the current source, and *A* is a constant coefficient. Then, the differential potential Eq. 2 becomes

4 $$ \varphi_{ij} = A \cdot \frac{I}{\sigma }\left| {\frac{1}{{r_{i} }} - \frac{1}{{r_{j} }}} \right| = A \cdot \frac{I}{\sigma }\frac{{\left| {r_{i} - r_{j} } \right|}}{{r_{i} r_{j} }} $$

In the upside quadrants, $\varphi_{{K^{'} N^{'} }} > \varphi_{{N^{'} M^{'} }} > \varphi_{{M^{'} C}} > \varphi_{CM} > \varphi_{MN} > \varphi_{NK}$. The two downside quadrants have the same distribution. If the measuring electrode N is disconnected, the potential at electrode N equals zero. At the same time, *φ*_*K*,*N*_ equals *φ*_*K*_ and might be saturated for the EIT system because its value is much higher than in normal conditions.

**Fig. 3:**
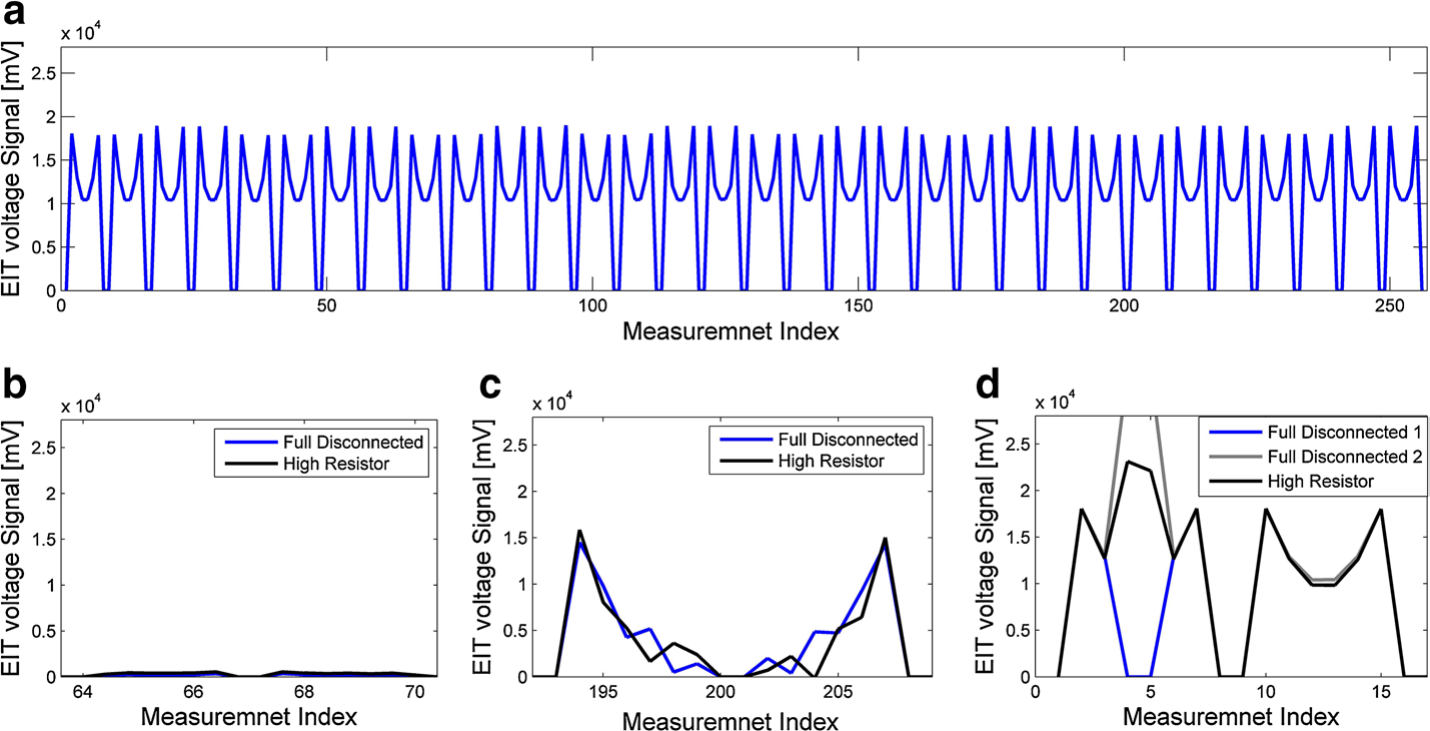
EIT data acquired on a resistor mesh phantom with a single-ended current source. **a** One frame of data is acquired with no disconnected electrode. Then, electrode 4 is disconnected by full disconnection and connecting a high value resistor. **b** Data acquired while electrode 4 acts as a positive excitation electrode. **c** Data acquired when the disconnected electrode 4 acts as negative excitation electrode. **d** Data acquired while electrodes 0 and 8 act as the drive pair and electrode 4 is disconnected: full disconnected 1 is the case in which the measurement including the disconnected electrode closed to a trivial value; full disconnected 2 is the case in which measurement is statured

To test the theoretical analysis, measurements were acquired with our EIT system on a resistor mesh phantom that simulates circular homogenous medium. The results are shown in Fig. 3. We simulated clinical disconnection by detaching the connection of electrodes or connecting a high impedance resistor in series. A $60\;{\text{k}}\Omega$ resistor reproduced measurements of actual clinical malfunctions well. When all electrodes are well connected, the boundary voltage is positively correlated with the distance between the measure electrodes and drive electrodes. Therefore, the measured data will have multiple saddle-shaped waveforms with similar levels of amplitude (Fig. 3). Then electrode 4 is set disconnected. The measurements when electrode 4 acts as the drive electrode follow the theoretical analysis. Once electrode 4 is included in the adjacent measuring pair, the measurement is statured or reduced to a trivial value because of the instable performance of the EIT system in such extreme disconnection cases. The measurement is abnormally higher in amplitude when the resistor is connected to electrode 4 [26]. Therefore, the results of theoretical analysis and verification of the resistor phantom are highly consistent. Regarding more complicated medium, such as the human head, the measurement performance also follows the consequences described above because of the constant current conduction principle.

**Fig. 4:**
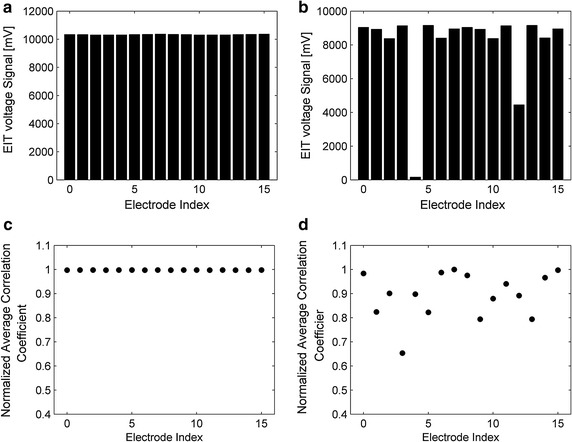
Mean values of cross-correlation coefficients and measurements recorded with each excitation. **a** Mean voltages of measurements corresponding to 16 excitations with no disconnected electrode. **b** Mean values of measurements corresponding to 16 excitations while electrode 4 is disconnected. **c** Normalized mean values of cross correlation coefficients corresponding to 16 electrodes with no disconnected electrodes. **d** Normalized mean values of cross-correlation coefficients corresponding to 16 electrodes while electrode 4 is disconnected

Through the theoretical analysis and resistor phantom tests, we determined that there existed a similarity among measurements with once current excitation. Correlation coefficient is one of the most widely used methodologies in similarity measures. We select the correlation coefficients combined with average measuring values to evaluate the electrodes. As shown in Fig. 4, the similarity of the invalid data is evaluated by correlation coefficients *corr*_*i*_ and the mean measuring value *ave*_*i*_ of data recorded under each current excitation. The *corr*_4_ and *ave*_4_ corresponding to disconnected electrodes are apparently lower than their values corresponding to normal electrodes. Thus, we proposed a method to determine disconnected electrodes based on these characteristics.

### Calculation of electrode variation coefficient

In this section, we propose a metric termed the electrode variation coefficient (EVC), which is obtained by calculating the weighted correlation coefficients for each electrode, and we determine disconnected electrodes by distinguishing abnormal EVC values.

**Fig. 5:**
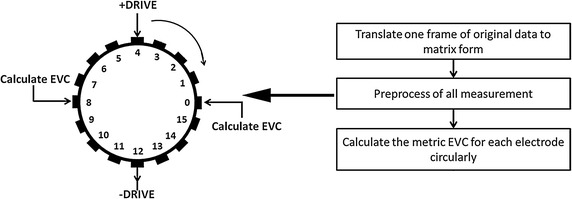
Schematic of the circulatory calculation of EVCs. With once current stimulation, only the EVC values of the two middle electrodes are calculated

In our method, only electrodes in the middle of the excitation pair are checked with each current excitation. Assuming electrodes 4 and 12 to be the excitation pair, the EVC values which correspond to electrode 0 and electrode 8 are calculated (Fig. 5). All EVC values are obtained by repeating this manipulation. We calculate the EVC values in this way because: First, measurements with different excitations have similar curve morphologies (Fig. 3), and therefore, the principle used to check one specific middle electrode is also able to detect other middle electrodes; Second, we only need to focus on the middle electrodes regardless of the actual location or number of disconnected electrodes because of the similarity. Via this process, we can simplify complicated scenarios with different numbers and locations of disconnected electrodes into limited cases.

The details of each step are given as follows:

(i) Calculate the correlation coefficients. Transfer one frame data vector $\varvec{d} \in R^{1 \times 256}$ into matrix form ${\mathbf{D}} \in R^{16 \times 16}$, where each column corresponds to 16 measurements in a single current excitation. The correlation coefficient matrix ${\mathbf{S}} \in R^{16 \times 16}$ is given by

5 $$ {\mathbf{S}} = \left[ {\begin{array}{*{20}c} {\rho_{00} } & {\rho_{01} } & \cdots & {\rho_{0n} } \\ {\rho_{10} } & {\rho_{11} } & \cdots & {\rho_{1n} } \\ \vdots & \vdots & \ddots & \vdots \\ {\rho_{n0} } & {\rho_{n1} } & \cdots & {\rho_{nn} } \\ \end{array} } \right](n = 15) $$

while

6 $$ \rho_{ij} = \frac{{cov\left( {X_{i} ,X_{j} } \right)}}{{\sqrt {DX_{i} } \cdot \sqrt {DX_{j} } }} $$

with *ρ*_*ij*_ as the correlation coefficient of the measured data with *i*th and *j*th excitation and *n* as the electrode number. The mean values of the correlation coefficients can be written as the vector

7 $$ {\mathbf{s}} = \left[ {s_{0} ,s_{2} , \ldots ,s_{15} } \right] $$

where

8 $$ s_{j} = \frac{1}{n}\sum\limits_{i = 1}^{n} {\rho_{ij} } $$

with *s*_*i*_ as the average value of correlation coefficients under the *i*th excitation.

(ii) Pre-process specific measured data. Compute the column means of matrix *D* and write it as the vector $\varvec{ave} = [ave_{0} ,ave_{1} , \ldots ,ave_{n} ]$. The element of $\varvec{ave}$ is calculated by

9 $$ ave_{j} = \frac{1}{n}\sum\limits_{i = 1}^{n} {D_{ij} } $$

According to the conduction characteristics of current flow in the head, the voltages obtained with target electrodes should be at a lower level compared with other measurements of the same-half under each current excitation. If the target electrode is disconnected, the corresponding measurement *D*(*i*, *j*) (*i* ∊ {3, 4, 11, 12}, *j* ∊ [0, 7]) will tend to a trivial value or an extreme value. In this step, we set the threshold *T*_*pre*,*j*_ defined as

10 $$ T_{pre,j} = (1 + a)ave_{j} $$

where *a* acts as a damping coefficient to adjust the metric of preprocessing in accordance with specific conditions. Here, *a* can be set to 0. Then, we set a threshold to normalize the affected voltages:

11 $$ D(i,j) = \left\{ {\begin{array}{*{20}l} {trivial} \hfill & {D(i,j) > T_{pre,j} } \hfill \\ {D(i,j)} \hfill & {D(i,j) < T_{pre,j} } \hfill \\ \end{array} } \right. $$

where *trivial* equals 10^−12^.

(iii) Calculate EVC values for all electrodes. In this step, we derive the proposed metric EVC by

12 $$ EVC_{i} = s_{k}^{ - 1} \times w_{i} $$

where *s*_*k*_ is the correlation coefficient of the *i*th electrode with *k* + *i* = 15 and *w*_*i*_ is the weighting factor for the *i*th electrode. The threshold *T*_*c*_ is set to select *w*_*i*_ by the following rules

13 $$ w_{i} = \left\{ {\begin{array}{*{20}l} {\left( {\frac{{D\left( {n,j} \right) + D\left( {n + 1,j} \right)}}{2}} \right)^{ - 1} } \hfill & {\frac{{D\left( {n,j} \right) + D\left( {n + 1,j} \right)}}{2} > T_{c} } \hfill \\ {\left( {{\text{ave}}_{i} } \right)^{ - 1} \times \mathop \sum \limits_{m = 0}^{m = 15} \frac{{{\text{ave}}_{m} }}{16}} \hfill & {\frac{{D\left( {n,j} \right) + D\left( {n + 1,j} \right)}}{2} < T_{c} } \hfill \\ \end{array} } \right. $$

Because in previous step the abnormal voltages were preprocessed, it is convenient to distinguish normal measurements from abnormal measurements. If there exists $\frac{{D\left( {n,j} \right) + D\left( {n + 1,j} \right)}}{2} > T_{c} {\kern 1pt} {\kern 1pt} {\kern 1pt} (j = [0,15],j \in Z)$, there are no disconnected electrodes.

### Detection of disconnected electrodes based on wavelet decomposition

Based on the EVC calculation method and the theoretical analysis above, we conclude that the EVC values of connected electrodes are consistent and tend to a trivial value, while the EVC values of disconnected electrodes tend to relatively high values. If the EVC values are sorted in ascending order, the EVC sequence of the connected electrodes is a relatively smooth and slowly varying parameter. Thus, the EVC sequence of disconnected electrodes reveals mutation points of high amplitude. In the signal analysis, such slowly changing signals are considered low frequency while dramatically changing signals are considered high frequency. Wavelet transformation is an effective tool for detecting the discontinuity points [8, 27]. The detailed decomposition coefficient at discontinuity points is rather high while the other coefficients spread around zero [28]. Therefore, the reconstructed detail coefficient would have higher amplitude at the discontinuity point, which is also the first point corresponding to disconnected EVCs. Therefore, we can localize the group of EVCs corresponding to disconnected electrodes by identifying the frequency discontinuity point in the ascending EVC sequence [27]. In our study, we used db4 wavelet function to perform the wavelet transform and to localize the discontinuity by detecting the minimum value.

Here, we apply discrete wavelet decomposition (DWT) to process the EVC sequence. We obtain the EVC and construct the EVC sequence as *x*_EVC_ = {*x*_*k*_ = EVC_*j*_}(*k* = 0, 1,…, 15) by sorting the EVC values in ascending order. Then, by implementing the fast wavelet transform algorithm [29, 30], the level one detail coefficients of the wavelet transform with the EVC sequence are given by

14 $$ d(l) = x_{EVC} (l) * g(l) = \sum\limits_{l} {g(l - k)x_{EVC} (l)} \quad {\kern 1pt} k = 0, \ldots ,2^{{j_{0} }} - 1 $$

where *g*(*l*) is the DB4 wavelet filter bank high-pass filter, and *j*_0_ is the coarsest level. *d*(*l*) was used to obtain reconstructed EVC sequence $\hat{x}_{EVC}$ by

15 $$ \hat{x}_{EVC} (l) = d(l) * g^{{\prime }} (l) = \sum\limits_{l} {g^{{\prime }} (l - k)d(l)} $$

where $g^{{\prime }} (l)$ is the reconstruction function for the detail coefficients.

If there are no disconnected electrodes, *x*_*EVC*_ will be consistent. If there are disconnected electrodes, there will be mutation points in *x*_*EVC*_ and minimum value in $\hat{x}_{EVC}$. As a result, we chose $\hat{x}_{EVC} (\hbox{min} )$ as the criterion of selection, electrodes with indices higher than the minimum are disconnected electrodes.

### Compensation algorithm based on grey model method

Dynamic brain EIT is a continuous process of data acquisition and monitoring, therefore, under normal circumstances the measured data has certain continuity [31–33]. For a particular time instants, the data can be regarded as the continuation of the previous period of data [34, 35]. The pre-data contain the potential changes of the monitoring object. Therefore, the data prediction method can be used to estimate the original data based on mathematical model calculated by using prior reliable measurements.

The grey systems theory was proposed by Deng in 1982 [36]. Grey prediction is an estimation of a grey system. Grey predication makes scientific, quantitative forecasts about the future output of a system by generating and extracting the useful information from a small number of samples and partially known information, which has a good application in the engineering field [37, 38]. The single variable first order grey model, which is abbreviated as GM(1,1), is the main and basic model of grey prediction. In this study, GM(1,1) is used to compensate for the invalid frames of data.

There are 4 main steps in this part: Generating the accumulation sequence, generating the reverse accumulation, establishing the grey model and data calculation. The detailed procedures are as follows:

(i) Generate the accumulation sequence. For one given measurement channel, the original data is expressed as *X* ^(0)^ = {*x* ^(0)^(1), *x* ^(0)^(2), …, *x* ^(0)^(*n*)}, where *n* is the sample size of data. Based on our experimental experience, we chose n = 30. The first-order accumulative generation converts *X* ^(0)^ to *X* ^(1)^ = {*x* ^(1)^(1), *x* ^(1)^(2), …, *x* ^(1)^(*n*)}, where *x* ^(1)^(*k*) = ∑ _*i*=1_^*k*^ *x* ^(0)^(*i*), *k* = 1, 2, …, *n*. Then, the adjacent neighbor mean sequence is computed as *Z* ^(1)^ = {*z* ^(1)^(2), *z* ^(1)^(3), …, *z* ^(1)^(*n*)}, where $z^{(1)} (k) = \frac{{x^{(1)} (k - 1) + x^{(1)} (k)}}{2}\;k = 2,3, \ldots ,n.$

(ii) Establish the first-order differential equation. Let *x* ^(1)^ be the solution of equation

16 $$ \frac{{dx^{(1)} }}{dt} + ax^{(1)} = u $$

where *a* and *u* are constant to solve out. If *t* = *t*_0_, the solution for *x* ^(1)^ = *x* ^(1)^(*t*_0_) is

17 $$ x^{(1)} (t) = \left[ {x^{(1)} (t_{0} ) - \frac{u}{a}} \right]e^{{ - a(t - t_{0} )}} + \frac{u}{a} $$

Letting *t*_0_ = 1, there is

18 $$ x^{(1)} (k + 1) = \left[ {x^{(1)} (1) - \frac{u}{a}} \right]e^{ - ak} + \frac{u}{a} $$

(iii) Establish the least square estimate sequence of the grey differential equation of GM(1,1). Discretize Eq. 16 to gain the GM(1,1) equation:

19 $$ x^{(0)} (k) + az^{(1)} (k) = u $$

Transform Eq. 19 to matrix form:

20 $$ y = BU $$

with

$$ y = (x^{(0)} (2),x^{(0)} (3), \ldots ,x^{(0)} (n))^{T} ,\;B = \left[ {\begin{array}{*{20}c} { - \frac{1}{2}[x^{(1)} (2) + x^{(1)} (1)]} & 1 \\ { - \frac{1}{2}[x^{(1)} (3) + x^{(1)} (2)]} & 1 \\ \vdots & \vdots \\ { - \frac{1}{2}[x^{(1)} (n) + x^{(1)} (n - 1)]} & 1 \\ \end{array} } \right],\;\;U = \left[ {\begin{array}{*{20}c} a \\ u \\ \end{array} } \right] $$

Solve the equation and obtain the values of coefficients with

21 $$ \hat{U} = \left[ {\begin{array}{*{20}c} {\hat{a}} \\ {\hat{u}} \\ \end{array} } \right] = (B^{T} B)^{ - 1} B^{T} y $$

(iv) Estimate the following data. Substitute $\hat{a}$ and $\hat{u}$ into *x* ^(1)^(*k* + 1) and get

22 $$ \hat{x}^{(1)} (k + 1) = \left[ {x^{(1)} (1) - \frac{{\hat{u}}}{{\hat{a}}}} \right]e^{\hat{a}k} + \frac{{\hat{u}}}{{\hat{a}}} $$

Set *k* = *m*, when *m* ≥ *n*, $\hat{x}^{(0)} (k + 1) = [\hat{x}^{(1)} (k + 1) - \hat{x}^{(1)} (k)]$ is the prediction of the original data sequence *x* ^(0)^.

There are 192 valid data channels in the EIT system. We need to repeat the above 4 steps 192 times to obtain a complete frame. Based on our experience in the trials, here *n* was set to 60, which indicates that 60 continuous frames are needed for the calculation.

To examine the reliability of the GM(1,1) model and the accuracy of predicted data, we used several parameters: the develop factor, the mean relative error (MRE), the mean posterior relative error (MPRE), the mean posterior correlation coefficient (MPCC). These parameters are defined as:

23 $$ {\text{MRE = }}\frac{1}{256} \cdot \frac{1}{\text{n}}\sum\limits_{j = 1}^{256} {\sum\limits_{i = 1}^{n} {\left| {\frac{{x_{j}^{(0)} (i) - \hat{x}_{j}^{(0)} (i)}}{{x_{j}^{(0)} (i)}}} \right|} } $$

24 $$ {\text{MPRE}} = \frac{1}{256}\sum\limits_{j = 1}^{256} {\left| {\frac{{x_{j}^{(0)} (i) - \hat{x}_{j}^{(0)} (i)}}{{x_{j}^{(0)} (i)}}} \right|} $$

25 $$ \begin{aligned} & {\text{MPCC}} = \frac{{\sum\nolimits_{i = 1}^{m - n} {(mean_{orign} (i) - mean_{1} )(mean_{predict} (i) - mean_{2} )} }}{{\sqrt {\sum\nolimits_{i = 1}^{m - n} {(mean_{orign} (i) - mean_{1} )^{2} } } \sqrt {\sum\nolimits_{i = 1}^{m - n} {(mean_{predict} (i) - mean_{2} )^{2} } } }}, \\ & mean_{1} = \frac{1}{m - n}\sum\limits_{i = 1}^{m - n} {mean_{orign} (i),} \quad mean_{2} = \frac{1}{m - n}\sum\limits_{i = 1}^{m - n} {mean_{predict} (i)} \\ & mean_{orign} (i) = \frac{1}{256}\sum\limits_{j = 1}^{256} {x_{j}^{(0)} (i)} {\kern 1pt} ,\quad mean_{predict} (i) = \frac{1}{256}\sum\limits_{j = 1}^{256} {\hat{x}_{j}^{(0)} (i)} {\kern 1pt} \\ \end{aligned} $$

The develop factor is −*a*, which is the indicator used to identify whether the grey model is suitable for long-term predictions. Here, *x*_*j*_^(0)^(*i*) is the *j*th voltage data of *i*th frame. ${\text{MRE}}$ evaluates the error of the grey model. The agreement of the predication and actual measurement is measured by parameters ${\text{MPRE}}$ and ${\text{MPCC}}$. Smaller the Develop Factor, ${\text{MRE}}$ values and ${\text{MPRE}}$ values correspond to larger ${\text{MPCC}}$ values, and better the prediction.

### Data acquisition procedure

To verify the effectiveness of the proposed approach, experiments were performed on a resistor phantom and clinical patients. EIT data were measured in real time using an EIT system (FMMU-EIT5). This system consists of 16 electrodes. The working frequency of the system ranges from 1 to 190 kHz, the current from 500 to 1250 μA with a measuring accuracy of ±0.01%. The common-mode rejection ratio is over 80 dB. We have carried out a series of experiments using this system and demonstrated is a reliable data acquisition system [34, 35]. A more detailed description of the EIT system is presented in previous studies [19, 20]. The reconstruction algorithm is damped least square method using finite element models [33]. In this study, 1 mA and 50 kHz altering current and 1 frame per second data acquisition speed was used. All calculation was implemented on a Pentium G630 computer.

**Fig. 6:**
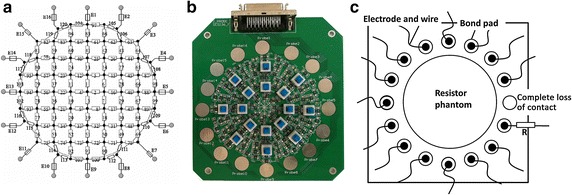
Illustration of the resistor phantom. **a** The topological graph. **b** The real photo. **c** Set-up of the disconnected electrodes on the resistor phantom

The physical model experiments were carried on a resistor phantom representing a circular homogeneous medium and comprising 120 resistors ($1\;{\text{k}}\Omega$) with 0.1% precision [39]. There were 16 unoccupied bonding pads and 1 SCIS-36 port on the phantom. Localized conductivity perturbations could be produced by operating the 16 push-type switches. The schematic of the resistor phantom is shown in Fig. 6. The finite element model for the physical phantom was a homogeneous circular mesh with 288 triangle elements. We simulated the disconnection by detaching the electrodes from the phantom or connecting high impedance resistor in series.

The clinical data were acquired at Xijing hospital, Fourth Military Medical University, Xi’an, China. They were approved by the Fourth Military Medical University Ethics Committee on Human Research and informed written consent was obtained from the patients’ relatives. In electrode detection scenario, six patients (five males and one female) were included. All patients were conscious and lay on the sickbed. Before the monitoring, sixteen copper cup electrodes were rigorously sterilized and placed with the conductive gel (Ten 20 conductive paste, Weaver and Company, Aurora, USA) on the circumference of the head. The set-up of disconnected electrodes was the same as in the phantom experiments. We simulated disconnection by detaching the electrodes from underneath of bandage around the head or connecting a resistor in series with the wire. In compensation scenario, data collected from the patients (two males) who received treatment of twist drill drainage in department of neurosurgery of Xijing hospital were analyzed retrospectively [34, 35]. The finite element model was obtained by segmenting the patient CT images into three parts (scalp, skull, parenchyma), which were further discretizing into 851 triangle elements. In both phantom and clinical trials, we selected a period of data to test the compensation method without setting disconnected electrodes.

## Results

### Detection of disconnected electrodes

**Fig. 7:**
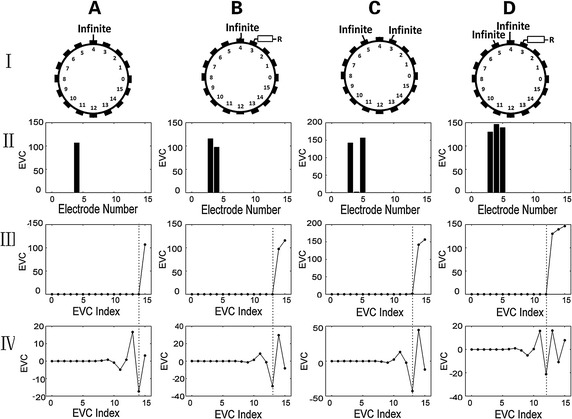
Results of the experiments on resistor phantom. Column A to D is the four different scenarios with different disconnected electrodes. Row I shows the detailed location of the disconnected electrodes. ‘Infinite’ and ‘R’ respectively represent complete loss of contact and the high impedance resistor connection. Row II presents the corresponding EVC values of all electrodes. Row III is the EVC values ordered from small to large, which are prepared to be processed with DWT. Row IV is the final results of the reconstructed EVC sequence with level one detail coefficients. In Rows III and IV, the *dashed lines* mark the location corresponding to the reconstructed EVC minimum and abnormal values in the original sequence

The resistor phantom experiments were carried out to validate the detection method. This experiment was performed to verify the correctness of the detection method rather than its limitations. The disconnected electrodes were simulated by total contact loss or by connecting a $60\;{\text{k}}\varOmega$ resistor in series with the electrode wire. We used ‘Infinite’ and ‘R’ to respectively represent complete loss contact and the resistor connection. Four different cases corresponding to data acquired with different distribution of disconnected electrodes are shown in Fig. 7. Column A is the basic scenario in which one electrode is disconnected. Column B and C illustrate the more complex scenarios with disconnected electrodes at different locations simulated by a total loss of contact and connecting a high value resistor. Column D shows three adjacent disconnected electrodes while two correspond to complete contact loss and one is connected to the resistor. Rows I to IV show the detailed outcome of each step. From Rows II and III, we can see that the EVC values of disconnected electrodes were much greater than normal ones. After the ranking process, the higher EVCs were moved to the right side of the sequence. By comparing Row III with Row IV, the mutation locations corresponded to the minimums of the reconstructed EVC signal, and the reconstructed EVCs behind the minimums in Row IV corresponded to the original abnormal ones. Therefore, using the minimums of the reconstructed EVC sequence, all abnormal EVC values were detected as well as the corresponding disconnected electrodes.

**Fig. 8:**
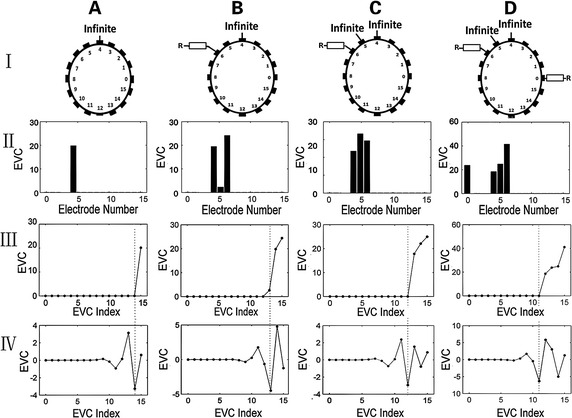
Results of disconnected electrode detection on patients. Columns A to D present four different scenarios of disconnected electrodes. Row I gives the detail of the disconnection distribution. ‘Infinite’ and ‘R’ respectively represent complete loss of contact and the high impedance resistor connection. Rows II to IV show the outcome of each step. In Rows III and IV, the *dashed lines* mark the correspondence between reconstructed EVC minimum and abnormal values in the original sequence

The results of the experiments on patients are shown in Fig. 8. The layout of the figure is the same as in Fig. 7 except that the max number of disconnected electrodes is increased to 4. The corresponding values of abnormal EVC in the reconstructed EVC sequence were behind the minimum marked by the dashed line in the graphs (Fig. 8 Row III and IV). By locating the minimums of the reconstructed EVC sequence, the EVC values of disconnected electrodes were filtered out.

Table 1 shows the robust performance evaluation of this detection method in real time monitoring during clinical experiments. The detection algorithm exhibited a high sensitivity for up to three disconnected electrodes. When there were four disconnected electrodes, the accuracy of the detection declined to 84%. In particular, if there were five disconnected electrodes, the algorithm could detect five electrodes in only 17% of cases. The results show that for more than four disconnected electrodes, it is difficult for this algorithm to accurately detect them.

**Table 1:** Performance evaluation of the detection algorithm in clinical condition

Number of disconnected electrodes	Number of clinical datasets tested	Number of correctly detected disconnected electrodes	Percentage of cases
0	12	0	100
1	12	1	100
2	12	2	100
3	12	2	8
		3	92
4	12	2	8
		3	8
		4	84
5	12	2	25
		3	33
		4	25
		5	17

### Compensation for invalid frames of data

**Fig. 9:**
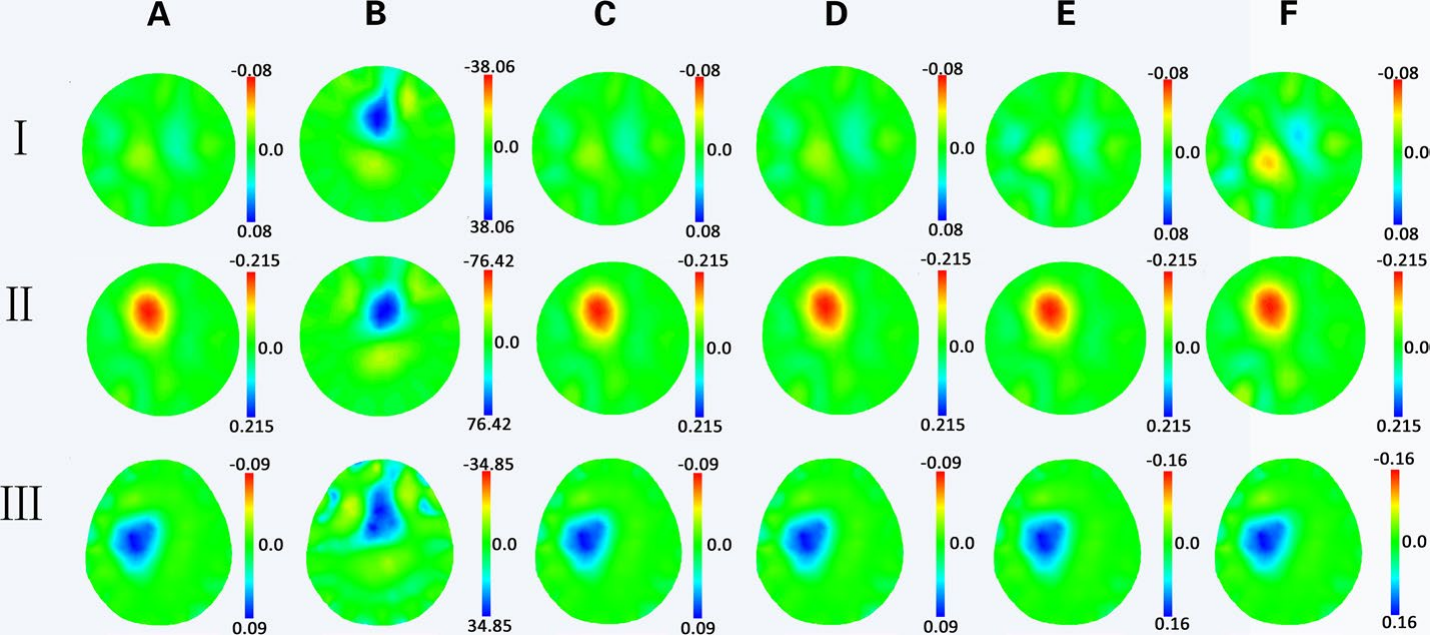
Comparison of the reconstructed images with the original data and predicted measurements. Rows I to III show three different experimental scenarios. In Row I, the data are acquired from a resistor with no target. In Row II, the data are acquired from a resistor with a target. In Row III, the data are acquired from clinical patients receiving twist drill drainage treatment. Column A is the original normal image. Column B is the image with electrode 4 disconnected. Column C and D are a comparison of the 1st image calculated with the original data and predicted after the instance of disconnection. Column E and F are a comparison of the 60th image calculated with the original data and predicted after the moment of disconnection

Figure 9 illustrates the effectiveness of compensation for invalid frames of data with resistor phantom and clinical patients. Because time differential images represent internal changes in conductivity between two instants, data acquired from the moment when no electrode is disconnected are used as a reference. In the experiments of resistor phantom, the target was created by shunting specific resistors. Figure 9 shows the results of reconstructed image. If there were disconnected electrodes, the images were seriously affected by artifacts (Fig. 9 Column A and B). By comparing Column C and D, the 1st frame image reconstructed by the compensated frame of data was capable of restoring the original image. Furthermore, we determined that this good performance could last for at least 60 frames by comparing the images in Column E and F.

Table 2 illustrates the accuracy of the grey model established with measured data and quantized characteristics of the estimated data. The Development Factor was quite low so that the grey model was suitable for long-term predications. It could also be found that the MRE are both under 1%, which meant that the grey model was qualified to make prediction. The MPRE was at a quite low level <0.01% while the MPCC was up to 0.88. Therefore, the estimated data were shown to be highly correlated with the original data.

**Table 2:** All parameters for comparison between the actual measurements and prediction

	Development factor	MRE	Model level	MPRE (1st frame)	MPRE (60th frame)	MPCC
Phantom without target	≤3.73e−04	≤0.0034	Qualified	≤3.2834e−06	≤3.2834e−06	0.8865
Phantom with target	≤4.67e−04	≤0.005	Qualified	≤1.7359e−05	≤1.7698e−05	0.8543
Clinical trial twist drill drainage	≤5.21e−04	≤0.0043	Qualified	≤2.2145e−06	≤6.5038e−06	0.7763

### Evaluation of time cost

**Fig. 10:**
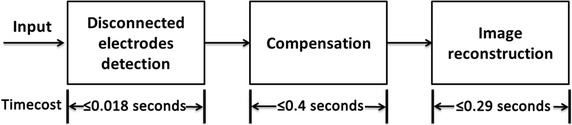
Illustration of time cost of the entire process. The disconnected electrodes detection completes calculations within 0.018 s. The compensation needs no more than 0.4 s. The entire calculation can be completed within 0.71 s

Figure 10 shows the details of the time cost of the entire process. The detection and compensation cost approximately 0.42 s at most. The compensation first required time to establish the GM(1,1) model, but it cost much less in the following prediction after establishing the grey model. Even with the calculation of the grey model, the time cost of the entire procedure, including detection, compensation and image reconstruction, could be restricted to <1 s. Such time cost is acceptable because the data acquisition speed is 1 frame per second.

## Discussion

This paper analyzed the problem of electrode disconnection during brain EIT monitoring in clinical environments, and we proposed a two-step approach to detect disconnected electrodes and to compensate for the invalid data. The experiments with data from the resistor phantom and patients proved an effective approach for managing such disconnections.

### Multiple disconnected electrodes detection

With the detection method, we can locate multiple disconnected electrodes with high accuracy to help medical staff to fix the disconnected electrodes and minimize the data loss as much as possible.

Different from previous studies, we defined the EVC values calculated from the weighted-correlation coefficients to evaluate the connection of electrodes and singled out the disconnected electrodes via DWT. In previous studies, the detection approaches were either based on the inverse and forward problem calculation or reciprocal principle theory. In Asfaw‘s method, detached electrodes were automatically detected through repeating forward and inverse calculations. He proposed that a set of ‘good’ electrodes could produce measurements consistent with each other and such consistency would be terminated if ‘bad’ electrodes were contained in the set, and thus erroneous electrodes could be excluded by verifying the consistency of the measured data with the electrode sets. However, the detection method required *n* × *n* (where *n* is the electrode number) calculations of the forward problem and inverse problem and thus was not suitable for real-time detection. Furthermore, the method was designed to reliably detect one detached electrode. In Hartinger’s method, faulty electrodes are confirmed by examining voltage-current reciprocal measurements. Although the detection method was reliable for its intended purpose, the sensibility was low in case of more than one faulty electrode. In addition, extra data were required if the data acquisition protocol of the EIT system cannot provide reciprocal measurements.

A result comparison with previous detection methods is shown in Table 3. Asfaw’s method shows potential in detecting multiple disconnected electrodes with a high accuracy. However, the computation time is too long to be applied to our data collection procedure. The accurate time cost of Hartinger’s method is not reported, but the main limitation of this method is that the accuracy of detection needs to be improved. Therefore, our method has a higher accuracy in detection with more disconnected electrodes. This method is applicable for our EIT system incorporating an opposite-drive adjacent-measure data acquisition protocol.

**Table 3:** Comparison of the results of two previous detection methods and the method reported in this study

Methods	Reported computing time	Reported detection number	Notes
Asfaw et al. [5]	About 4 s	Designed for 1	Scenarios for 2 and 4 disconnected electrodes were shown
Hartinger et al. [4]	Real-time, accurate time is not reported	1 with 100% accuracy 2 with 96% accuracy 3 with 8% accuracy	
The presented method	within 0.018 s	1 or 2 with 100% accuracy 3 with 92% accuracy 4 with 84% accuracy	

In addition, Ghanem implemented wavelet decomposition to detect lead failure of ECG electrodes [8]. Two parameters were extracted from the reconstructed approximation sequence and reconstructed detail sequence to build a no lead failure zone. If the point with the two parameter as X and Y coordinates falls in the lead failure zone, there is a lead failure in the acquired data which is processed by wavelet decomposition. Unfortunately, this implement is not suitable for brain EIT because EIT data has no significant spikes to extract and such detection might be hysteretic. Ross developed a system and method for correcting fault conditions in soft-field tomography [9]. His projection detected fault excitation through mismatch response and compensated the valid data by a pre-calculated output. However, the successfulness relied on the redesign of hardware and therefore, not as practical as our method.

In the detection method, we use correlation coefficient to measure the similarity. This similarity can be represented by other statistical parameters, such as Euclid distance, Hausdorff distance, cosine distance and dynamic time warping [40–43]. These parameters are used to measure sequence similarity in many cases. However, the performance of the Euclid and Hausdorff methods may introduce issues in comparing between measurements from different frames because their calculations of absolute distance highly depending on the calculation baseline. The cosine distance focuses more on the direction change of the data sequence, not the shape of the curve. Dynamic time warping introduces more calculations and does not exhibit an apparent advantage over correlation coefficients [42]. To avoid potential problems and to cooperate with the following calculation, we selected the correlation coefficients. Besides, we utilize wavelet decomposition to filter out abnormal EVC values corresponding to disconnected electrodes. Here, we primarily take advantage of the time–frequency correspondence characteristic of the wavelet transform. Other time–frequency methods include the Hilbert transform, the short time Fourier transform and the quadratic time–frequency distribution [44–47]. These methods are also used to extract instantaneous characteristics of the signal. However, we need a very precise one-to-one correspondence between the original EVC and the reconstructed EVC with the detail coefficients. The wavelet transform has the ability to provide high time resolution in high frequency to meet our requirements [48, 49]. Other methods do not offer such point to point accuracy.

During the experiments, to verify the effectiveness of the disconnected electrodes detection, we disconnected multiple adjacent electrodes to simulate various possible scenarios. With data acquired from each excitation, we only checked the two middle electrodes between the current-driving electrode pair. According to Eq. 2, the amplitude of the measurement containing the middle electrode is usually less than other measurement on the same side, which makes it easier to preprocess the abnormal measurement. By locking the relative position of the target electrode, we simplify all possible scenarios of disconnection into four cases: ‘good’–‘bad’–‘good’, ‘bad’–‘bad’–‘good’, ‘bad’–‘good’–‘bad’, ‘bad’–‘bad’–‘bad’, while the middle electrode is the target to check. This design greatly decreases the logical complexity and location sensitivity. Experiments on the resistor phantom showed that all disconnected electrodes could be filtered out with 100% accuracy. However, due to noise and target differences between clinical and laboratory environments, we did not further evaluate the phantom tests after verification of the methodology principle. In clinical trials, before the monitoring was started, it should be ensured that all electrodes are well-connected. The results from patients showed that the detection method was able to achieve very high accuracy if the number of disconnected electrodes is no more than four. However, disconnections would lead to interference in the measurements made by the data acquisition system in an ICU environment. Therefore, in several scenarios in which less than five electrodes were disconnected, the detection sensitivity was not able to reach 100%. Moreover, if there are five or more disconnected electrodes, the weighting part for calculating EVCs becomes very unstable and might affect the use of the minimum of wavelet reconstruction to determine abnormal EVC values, which would lead to missed disconnected electrodes. In the clinical experiments, more complicated explorations with more disconnected electrodes were not performed because other ‘good’ electrodes could be compromised when adjusting the disconnected electrodes if there are more than four disconnected electrodes. Therefore, removing the bandage to examine and fix all electrodes is a more reliable way to eliminate such disconnections. Otherwise, this detection principle of this method is not only suitable for EIT systems with opposite-drive adjacent-measurement protocol, but also suitable for systems with pseudoopposite-drive adjacent-measurement protocol or other working protocols.

The electrode disconnections actually reflect malfunctions of data acquisition system. The most common numerical simulation method is not applied in this paper. The numerical simulation could display the voltage and current distribution of the imaging area, but for electrode disconnection, the affected data are not inexistent but missed in the transmission from the body to the EIT system through electrodes. The numerical simulation results are not related to the peripheral hardware. Therefore, we chose to show simulations on resistor phantom.

If an electrode is of incomplete contact, the contact impedance will increase compared with a well-connected electrode. The contact impedance affects the EIT measurement in two ways. First, contact impedance affects the current distribution beneath the electrode inside the body [14]. Second, contact impedance causes the common mode voltage to yield a differential mode voltage at the amplifier input of EIT system [26]. The incomplete contact introduces interface into the voltage measurement through these two ways described above, which leads to artifacts in the image reconstruction. In circumstance of incomplete contact, current is still able to flow through the electrode. But in disconnection there is little drive current flow through the electrode into the body. So the voltage measurements from disconnected electrode become unstable, and the measurements acquired are different from the connected electrode in amplitude and curve shape, when the disconnected electrode acts as positive electrode or negative electrode.

The aim of detecting disconnected electrodes is to reduce data loss. In this study, the detection method presented in this study makes a judgment as to whether the electrode is disconnected or not. The method does not quantitatively reflect the contact status of the electrodes. Recent studies on EIT electrode primarily focus on electrode scenarios with incomplete contact, where there is still normal current injection, and the measurement error mainly comes from current distribution distortion caused by electrode–electrolyte layer [11, 13, 50]. Mamatjan et al. [51] proposed a method to quantitatively evaluate EIT data quality. The method provided an overall assessment of the whole dataset. In next step, we will evaluate each single electrode with detailed scenarios regarding where the current distribution is affected or the common mode error is included, because these issues are not clearly addressed at the present.

### Compensation for invalid frames of data

In the compensation part, we employed the GM(1,1), which was established with normal measurements before disconnection, to predict the subsequent frames of data. The predicted data can be used to reconstruct images without disconnection artifacts. In previous studies, the compensation was based on reconstruction algorithm improvement [3, 4]. Adler and Hartinger both modified the measurement noise covariance matrix in the MAP reconstruction algorithm to compensate for invalid data. And they still required rest valid data to continue monitoring. Our prediction method does not need to recalculate the reconstruction matrix.

We made full use of the valid data before the disconnection occurred to establish GM(1,1) to predict the data to replace the invalid frames. Indeed this method could not utilize the residual valid data. However, in most circumstance of long-term monitoring, it is not possible to have intracranial pathological or physiological changes, which will lead to a dramatic change in the brain impedance in minutes. This means that the latter measurement could be considered an extension of the previous measurement. Therefore, it is reasonable to extract the features of existing valid data to predict the subsequent measurements in specific brain EIT monitoring. Our compensation method offers another thought that based on available frames of data rather than the reconstruction algorithm improvement to compensate the data loss.

The result of grey model compensation depends on the data used to establish the grey model. If the interval of two interruptions is shorter than the data frames that we need to establish grey model, the effect of compensation will be affected. If the contact is lost momentarily, the compensation method will start to compensate for invalid frames of data and the compensation procedure will not until all electrodes are connected. Whether the compensation is activated or inactivated depends on the disconnected electrode detection result. There is one scenario that the contact is lost immediately after the monitoring begins, the compensation algorithm could not compensate the lost data. Because we need a period of good data before disconnection happens to establish the grey model. However, the dynamic brain EIT monitoring is a long-term process, so the data loss at the beginning of monitoring has limited effect on the overall monitoring results.

One limitation of compensation algorithm is that it couldn’t utilize the residual valid data. But in most circumstance of long time monitoring, it is not possible to have intracranial pathological or physiological changes, which will lead to a dramatic change of the brain impedance in minutes [42, 43]. So although there are some limitations, the compensation method could meet our need.

After electrode disconnection happens, the reconstructed image will be invalid immediately. The grey model compensation is designed to offer the predicted image result until the clinical staff comes to solve the issue. Afterwards, a new reference frame will be established. The image result before disconnection cannot be shown in the new image. If we continue to monitor without reselecting reference frame, the position and contact status of re-adhering electrodes are changed compared with their initial conditions and artifacts will be introduced in monitoring image [3]. Therefore, in further research, we need to consider how to inherit the information of former image into new monitoring images by combining present techniques.

## Conclusions

In clinical long-term dynamic brain EIT monitoring, electrode disconnections are a common occurrence and will lead to a failure of data acquisition. This paper offers a two-step solution to address the electrode detection. Our approach is able to detect more disconnected electrodes with a higher accuracy. The invalid frames of data were replaced by calculating a grey model with more stability. This proposed method is based on the features of available measurements rather than improving hardware or reconstruction algorithm in previous studies, which offers a novel way to deal with such problems.

## Abbreviations

EIT: electrical impedance tomography
MAP: maximum a prior
EVC: electrode variation coefficient
DWT: discrete wavelet decomposition
GM: grey model
MRE: mean relative error
MPRE: mean posterior relative error
MPCC: mean posterior correlation coefficient
